# Hypoglycemic Encephalopathy

**DOI:** 10.1002/ccr3.72453

**Published:** 2026-04-01

**Authors:** Dou dou Li, Bao‐ping Xu, Ba‐yi Liu, Hua‐min Wang, Hao Yang

**Affiliations:** ^1^ Department of Critical Care Medicine Zhongshan Hospital of Traditional Chinese Medicine affiliated to Guangzhou University of Traditional Chinese Medicine Zhongshan Guangzhou China; ^2^ Department of ICU, Dongzhimen Hospital Beijing University of Chinese Medicine Beijing China

**Keywords:** alcohol poisoning, hypoglycemia, hypoglycemic encephalopathy, magnetic resonance imaging

## Abstract

Hypoglycemic encephalopathy (HE) is a critical condition that can be challenging to early diagnose early; it can cause long‐lasting mental changes, disability, and even death. Herein, we describe a case of a 59‐year‐old male patient with HE occurring in acute alcoholism, the MRI, especially diffusion‐weighted imaging, showed diffuse high‐signal intensity in the bilateral basal ganglia, cerebral cortex, periventricular region, centrum semiovale, and corpus callosum.

## Key Clinical Message

For patients with altered mental status, monitoring fingertip blood glucose is necessary to rule out hypoglycemia as a cause. Hypoglycemic encephalopathy can present with extensive, symmetric MRI abnormalities. Early recognition, particularly on diffusion‐weighted imaging, is essential, as prompt correction of hypoglycemia may limit neurological damage.

**FIGURE 1:**
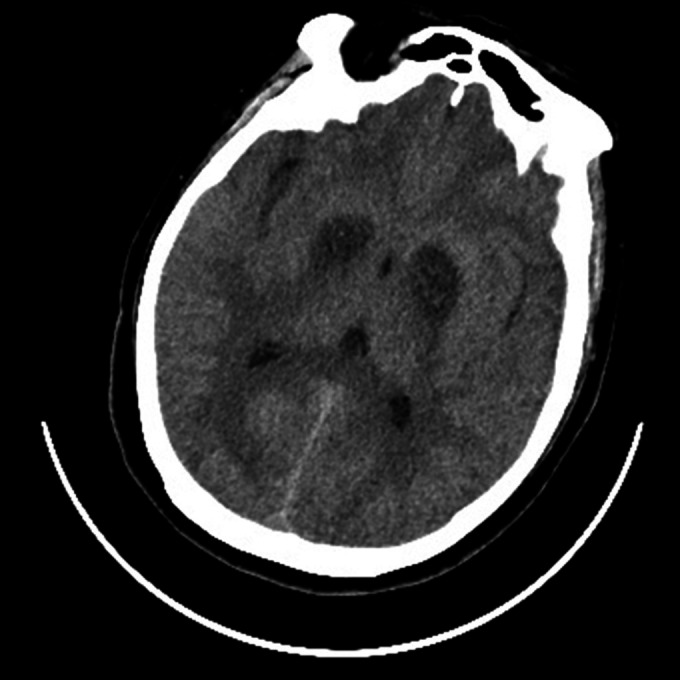
Axial computed tomography scan of the brain showed symmetrical hypoattenuation of the bilateral basal ganglia.

**FIGURE 2:**
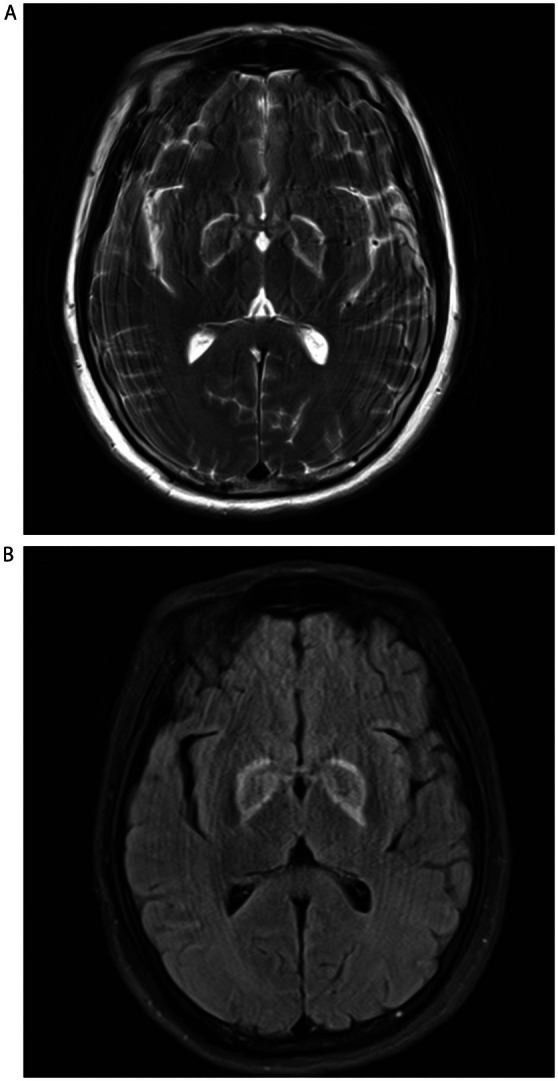
Brain T2‐weighted magnetic resonance imaging demonstrated symmetrical high signal intensity and slightly longer T2 signals in the bilateral basal ganglia, corpus callosum body and periventricular, and centrum semiovale (Figure 2A); the diffusion‐weighted imaging sequences revealed specific symmetrically higher signal lesions in the bilateral basal ganglia, corpus callosum, cerebral cortex, periventricular, centrum semiovale and hippocampus (Figure 2B).

A 59‐year‐old man with no history of disease was found unconscious at home 12 h after he had last been seen. The patient had ingested a considerable quantity of alcohol the previous night, estimated to contain approximately 300 g of ethanol. On admission, his vital signs at the emergency department were normal, and his blood glucose level was 30.6 mg/dl. Cerebrospinal fluid analysis showed a normal cell count and protein level. Cytologic and microbiologic investigations including serum thiamine (vitamin B1) concentration had negative results, as did investigations for other metabolic and toxic causes; Wernicke encephalopathy and feeding disorders were also ruled out. Whereas axial computed tomography (CT) scan of the brain showed symmetrical hypoattenuation of the bilateral basal ganglia (Figure 1). Diffusion‐weighted magnetic resonance imaging (MRI) of the brain revealed extensive symmetrical hyperintense lesions in the corpus callosum body, bilateral basal ganglia, periventricular, and centrum semiovale, which did not conform to cerebral arterial distributions (Figure 2). Based on these results, we diagnosed hypoglycemic encephalopathy (HE), likely resulting from prolonged episodes of hypoglycemia. Brain T2‐weighted MRI demonstrated symmetrical high signal intensity and slightly longer T2 signals in the basal ganglia, periventricular, and centrum semiovale (Figure 2A), the diffusion‐weighted imaging (DWI) sequences revealed specific symmetrically higher signal lesions in the bilateral basal ganglia, cerebral cortex, periventricular, centrum semiovale and hippocampus (Figure 2B). Two months after admission, the patient was discharged from the hospital by his family.

HE may cause diverse clinical manifestations, ranging from systemic and focal neurological deficits to irreversible coma, and there are no special signs in early head CT, which is often misdiagnosed as stroke or other acute neurological diseases [1]. Imaging can provide early clues for clinical suspicions and play an important role in diagnosis, assessment of the response to therapy, and prognosis [2]. MRI is a sensitive technique used to evaluate HE at an early stage, especially DWI sequences; it can help narrow the differential diagnosis and improve the prognosis of patients [3]. MRI of HE usually reveals specific symmetrical high‐signal lesions in the bilateral basal ganglia, cerebral cortex, substantia nigra, corpus callosum, and hippocampus [2]. The pathological degeneration includes some combination of selective neuronal death, proliferation of astrocytic glial cells, paramagnetic substance deposition, and/or lipid accumulation, which may suggest the particular susceptibility and high energy demand of these areas to hypoglycemia in the human brain [2]. Unlike stroke MRI findings, these lesions usually do not conform to arterial distribution and can be reversed by normalizing blood sugar levels [2]. The prognosis of HE depends on the severity, duration and condition of the organism. Despite intravenous glucose injection and drip being performed repeatedly, due to the long duration of hypoglycemia, the overall prognosis of the patient was poor.

## Author Contributions

**Dou dou Li:** conceptualization, writing – original draft. **Bao‐ping Xu:** conceptualization, writing – original draft. **Hua‐min Wang:** data curation, project administration. **Ba‐yi Liu:** data curation, project administration. **Hao Yang:** writing – review and editing.

## Funding

The authors have nothing to report.

## Ethics Statement

Written informed consent for publication of their clinical details and/or clinical images was obtained from the patient.

## Conflicts of Interest

The authors declare no conflicts of interest.

## Data Availability

The data were available upon appropriate requests from the corresponding author.
